# Supplementary figures and images for: Molecular Analysis of Anti-Tuberculosis Drug Resistance of Mycobacterium tuberculosis Isolated in the Republic of Korea
Source: Antibiotics (Basel). 2023 Aug 17;12(8):1324. doi: 10.3390/antibiotics12081324 (PMC10451913; doi:10.3390/antibiotics12081324)

## Slide 1
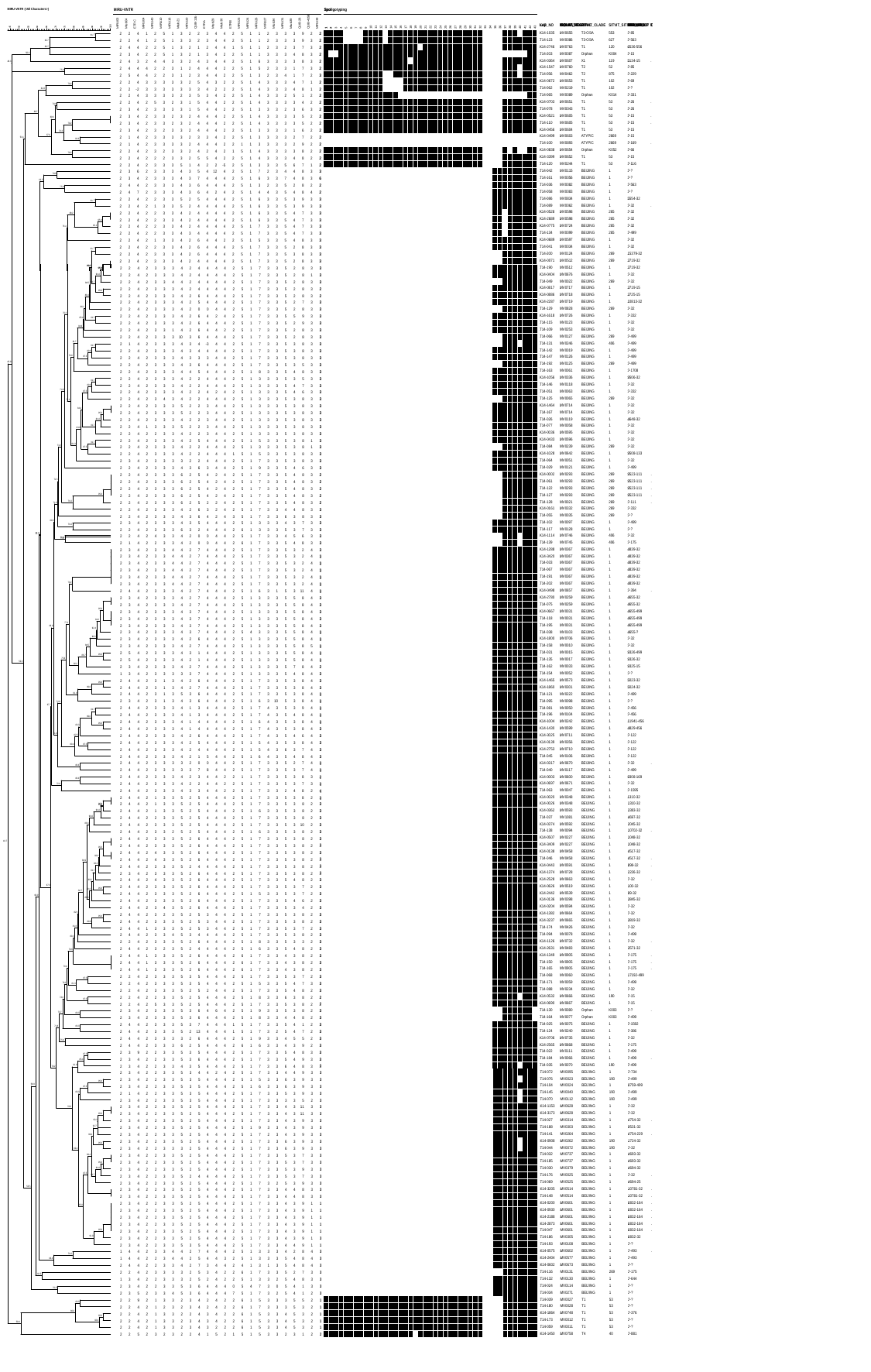

Supplement: Supplementary file 1 [file antibiotics-12-01324-s001.zip › Supplementary Data_Figure S1.pptx]
